# A Woman's Sacrifice.—For Duty's Sake

**Published:** 1887-09-17

**Authors:** 


### A Woman's Sacrifice.—For Duty's Sake.

SINCE the Royal Infirmary, Edinburgh, removed to the new buildings opposite the University, great changes for the better have been made in its administration. Experts who have visited this institution of late years have one and all been struck with the excellence of the internal economy and management. Great credit is due to Surgeon-General Fasson for what he has done during the time he has been Superintendent, and he would be the first to acknowledge that Miss Pringle's devotion and capacity have brought about changes to which much of the present efficiency must be credited. Miss Pringle is devoted to Miss Nightingale, and her every wish is a law too sacred to be set aside or disregarded. Able, decided, and skilful herself, she still reveres the chief whom she followed as a novice.

When the Governors of St. Thomas's Hospital were compelled to look for a matron in consequence of Mrs. Wardroper's much to be regretted resignation, they came to the conclusion that Miss Pringle was in all respects the most suitable successor that could be found. Miss Pringle was, however, settled in Edinburgh, where she was surrounded by every comfort, in the receipt of a larger income than she would receive at St. Thomas's, and at the head of a trained and capable staff who were devoted to her, and to whom she was bound by ties of long association and sympathy. There were, indeed, a multitude of reasons—official, personal, pecuniary, and social—why Miss Pringle should desire to remain in Edinburgh, and few, if any, to induce her to come to St. Thomas's. In these circumstances it is not a little noteworthy that Miss Pringle should have decided to put aside all these personal and pressing reasons, and to come to St. Thomas's to undertake a difficult and new responsibility because it has been represented to her that duty demands this sacrifice at her hands. It is one thing to be a hero amid the excitement of the battle-field, and quite another thing to be heroic in the discharge of every-day duty. It is, we believe, a fact that no calling produces more heroes and heroines, who pass unrecognised to their graves, than the medical and nursing professions. And, after all, those who do noble acts for duty's sake usually desire no other recognition than the sense that they have done their best to do what was right under trying circumstances and at all times. Miss Pringle, we are sure, for instance, would be the last person in the world to wish or sanction any recognition of the act of self-denial which it has been our pleasing duty to here record. Still, such acts ought to be published for the encouragement of others, and as a just tribute to the members of a noble profession, which includes in its ranks many of the best women of our day and generation.

